# Personalized Mobile Health for Elderly Home Care: A Systematic Review of Benefits and Challenges

**DOI:** 10.1155/2023/5390712

**Published:** 2023-01-16

**Authors:** Shahrbanoo Pahlevanynejad, Sharareh R. Niakan Kalhori, Meysam Rahmani Katigari, Rahil Hosseini Eshpala

**Affiliations:** ^1^Department of Health Information Technology, School of Allied Medical Sciences, Semnan University of Medical Sciences, Semnan, Iran; ^2^Department of Health Information Management, School of Allied Medical Sciences, Tehran University of Medical Sciences, Tehran, Iran; ^3^Peter L. Reichertz Institute for Medical Informatics (PLRI), Technical University of Braunschweig and Hannover Medical School, Braunschweig, Germany; ^4^Health Information Technology Department, Saveh University of Medical Sciences, Saveh, Iran; ^5^School of Allied Medical Sciences, Tehran University of Medical Sciences, Tehran, Iran

## Abstract

Mobile health as one of the new technologies can be a proper solution to support care provision for the elderly and provide personalized care for them. This study is aimed at reviewing the benefits and challenges of personalized mobile health (PMH) for elderly home care. With a systematic review methodology, 1895 records were retrieved by searching four databases. After removing duplicates, 1703 articles remained. Following full-text examination, 21 articles that met the inclusion criteria were studied in detail, and the output was presented in different tables. The results indicated that 25% of the challenges were related to privacy, cybersecurity, and data ownership (10%), technology (7.5%), and implementation (7.5%). The most frequent benefits were related to cost-saving (17.5%), nurse engagement improvement (10%), and caregiver stress reduction (7.5%). In general, the number of benefits in this study was slightly higher than the challenges, but in order to use PMH technologies, the challenges presented in this study must be carefully considered and a suitable solution must be adopted. Benefits can also be helpful in persuading individuals and health-care providers. This study shed light on those points that need to be highlighted for further work in order to convert the challenges toward benefits.

## 1. Introduction

Population aging is a global phenomenon that has affected the entire world [1]. The United Nations reports on population indicate that today, the median age for the world is 28 years. By 2050, it will likely increase by 10 years to reach 38 years. From 1950 to 2009, the global population of people over the age of 65 years increased from 8 to 11%, and it is expected to reach 22% by 2050 [1, 2]. This has many consequences on social care, health, retirement, housing, transportation, and economic performance [3].

The elderly are prone to the adverse side effects of various chronic diseases such as hypertension, cardiovascular disease, diabetes, and dementia [1, 4, 5]. One of the most important challenges in different countries is the subject of population aging consequences. Preparing for old age is part of health and infrastructure policies for the social and economic development of countries [6]. In the United States in 2010, people over the age of 65 needed healthcare at a cost three times higher than that of the average adult of working age and about five times higher than that of the average child [7]. Population aging significantly increases the cost of healthcare, especially in the management of chronic diseases [8, 9]. Even in advanced health-care systems, the annual cost of diagnosing and treating chronic diseases is significantly increasing [10]. In recent years, due to increasing disease burden and costs especially among the elderly, the provision of medical and nursing services has shifted from hospitals to homes, known as home care [11]. The purpose is to reduce hospitalization and transportation costs, improve the quality of healthcare by reducing medical errors, and ultimately enhance the independence and interaction of patients at home [12, 13].

Mobile health (m-health) as one of the new technologies can be a proper solution to improve the quality of nursing care, enable remote visits, and reduce health-care costs, thereby promoting the elderly's empowerment, improving monitoring, supporting healthcare at home, and preventing chronic diseases [7]. Mobile health refers to mobile telecommunications technologies that promote health through the provision of healthcare [14, 15]. This smartphone can become the medical hub of the future and has many capabilities in personalizing services [14]. Mobile-app-based health interventions may be an effective strategy for improving health-promoting behaviors in the disease-free general population [16]. Personal medicine is defined as the adaptation of medical treatments to the specific characteristics of patients [5, 6, 17]. In this approach, health providers consider treatments and interventions for individuals, taking into account the heterogeneity of diseases and external factors such as the environment, patients' needs, and different lifestyles [18]. It is more accurate in predicting risk and responding to treatments than traditional medicine [14, 19]. Studies have shown that mobile technology can play an important role in achieving this new approach in medicine [14].

Mobile apps provide an easy way to access the target group and are cost-effective compared to phone-based and clinic-based interventions [20, 21]. Currently, mobile phones are an important as well as a popular communication tool throughout modern society.

Despite the many benefits of m-Health in promoting care for the elderly and assisting with personalized health-care services, it is still important to explore all aspects of the issue and become familiar with the benefits and challenges that may affect the health of the elderly [22]. Identifying these plays an important role in promoting care for the elderly, reducing barriers, and increasing the positive points and benefits of these systems. Without careful consideration, not only may the elderly not enjoy the benefits of these systems but they may face major challenges. As a result, we must first identify potential benefits and challenges and then help to improve the health system by planning, designing roadmaps, and providing solutions to potential challenges [23].

Previous studies have typically focused on how to use m-Health for elderly care without focusing on personalized m-Health, its benefits, and challenges [17, 24–27]. Most studies are aimed at assessing the effects of technology-based activities on memory, communication, or engagement [16]. Mobile apps based on their skills training and cognitive-behavioral therapy principles are promoted by health-care systems (such as Veterans Affairs Medical System and Kaiser Permanente) and marketed directly to consumers [28]. A large-scale study found that adopting mindfulness practices among subscribers helped alleviate mental health symptoms during the pandemic. Another study found that apps can increase treatment engagement for people who are unwilling to participate in traditional mental health and substance use services [29]. There is evidence of increased adverse health situations during the COVID-19 pandemic and digital health and mobile apps were used to work as solutions [30]. Digital health solutions emerged in popularity even before and during the pandemic and appeared as a viable strategy for reaching people with limited access to health services [31]. In the context of COVID-19, there has been a considerable shift in telehealth capabilities [32, 33]. In order to benefit from available mobile-based systems practically, it is required to understand their pros and cons in theory, particularly in a personalized version which is a more upgraded version. Therefore, this study is aimed at reviewing the benefits and challenges of personalized mobile health (PMH) for elderly home care.

## 2. Materials and Methods

### 2.1. Search Strategy

An electronic search of three scientific databases (Scopus, Web of Science, and PubMed) was conducted on January 4, 2021. The search strategy was formulated based on four core concepts: “personalized medicine”, “self-care”, “elderly”, and “M-health AND telehealth”. The search was not limited. We adopted the PICO approach to prepare the search terms [34].

Combinations of the following search terms (A to D) were used:

- Personalized medicine: Precision Medicine OR Individualized Medicine OR Personalized Medicine OR targeted OR tailored OR Personalized∗
- Self-care: follow∗ OR monitor∗ OR surveillance OR care∗ OR self-management OR self-management OR self-care OR self-care OR self-treatment OR self-treatment OR Home Care Services
- Elderly: aged OR senil∗ OR geriatric OR elder∗
- M-Health AND tele-health: wearable OR m∗health OR health app∗ OR mobile health app∗ OR mobile medical app∗ OR medical smartphone app∗ OR mobile OR smartphone OR portable software app OR portable electronic app OR PDA OR computer palmtop OR personal digital assist∗ OR palm pilot OR pocket pc OR tablet computer OR computer handheld OR telemedicine

Then, we used MeSH, Emtree, and other related papers to find all the keywords related to these categories. First, a standardized search strategy was performed in PubMed; then, in other databases, then this strategy was modified according to the specific symbols and search methods in that database to obtain the most relevant related results. The steps for formulating the search query for PubMed are presented in Table 1.

### 2.2. Inclusion Criteria

The search for challenges and benefits for elderly was restricted to the English language and journal articles. Articles would be included if they reported the personalized mobile technology application challenges and benefits in elderly home care. As technology advances quickly, and the challenges and benefits faced by these technologies differ over time, to investigate recent challenges and benefits, this study included only articles published in the past 10 years (2010–2021 inclusive).

### 2.3. Exclusion Criteria

As this study is focused on reviewing personalized mobile technology application challenges and benefits in elderly home care, articles that studied nonhuman monitoring; focused on non-clinical purposes; were conference proceedings; were not mobile-based (CD, telephone, VR (virtual reality), email, video, SMS, robot, portal, media, and computer-based); were unrelated; were duplicated; had unavailable full texts or were abstract-only studies; were of other types, e.g., reviews; and systematic reviews articles that were entered in the search result were excluded.

### 2.4. Screening and Article Selection

The articles were imported into the EndNote reference manager software 20. After the removal of duplicates, to select studies, they were screened independently for their titles, abstracts, and a full-text review by two independent reviewers. Records of potentially relevant articles were then downloaded and imported into EndNote for eligibility assessment. Studies were eligible for data extraction if they met all the inclusion criteria and did not meet the exclusion criteria in the opinion of the reviewers. Disagreements between the reviewers were resolved by reaching a consensus or by consulting a third reviewer. Disagreements were resolved by discussion between the reviewers until consensus was achieved. If disagreements persisted, a third reviewer made the final decision. Unclear or missing information was identified, and the corresponding authors of each review were contacted with a request to provide the information. The AMSTAR 2 scores [35] were shared with the corresponding authors of the individual reviews to avoid any possible misinterpretation and to confirm the scores awarded.

### 2.5. Risk of Bias Assessment

Because in this research our purpose was to provide an analysis of articles and studies available for elderly home care in personalized mobile applications and evaluate their overall challenges and benefits, we did not intend to conduct a meta-analysis on the data; however, we used the AMSTAR 2 tool to perform a quality assessment of the included studies.

### 2.6. Analysis

According to the objectives, the extracted information was tabulated, categorized, and presented graphically in a hierarchical structure. The data were categorized using a bottom-up approach to reach a classification of the most general challenges and benefits. This review is reported according to the Preferred Reporting Items for Systematic Reviews and Meta-Analyses (PRISMA) guidelines [36].

## 3. Results and Discussion

**Figure 1:**
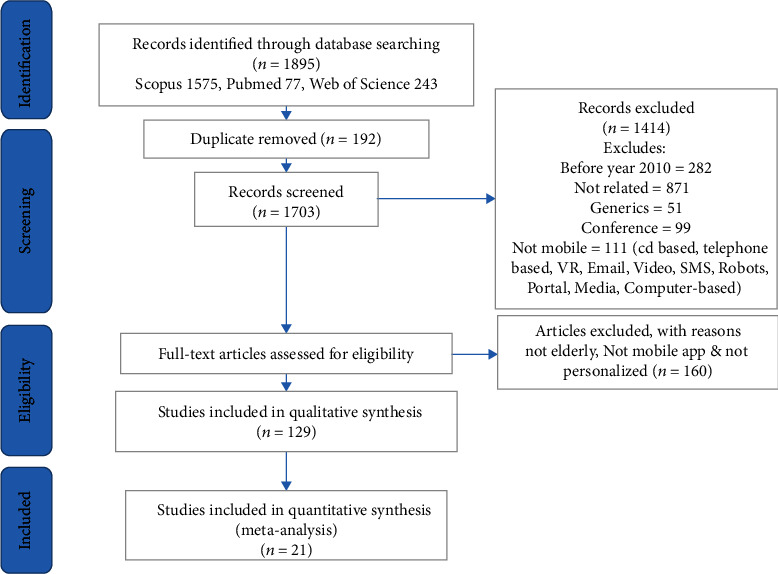
The PRISMA-based article selection process flow chart.

Initially, 1895 records were retrieved by searching the formerly mentioned databases. After removing duplicates, 1703 articles remained. Based on a review of titles and abstracts, 129 were found to meet the initial selection criteria. After an examination of the full texts, 21 articles met the inclusion criteria and were included in the final review. The process of article selection is shown in Figure 1.

### 3.1. PRISMA

Finally, 21 studies were included in this systematic review. The challenges and benefits extracted from these 21 articles are presented in brief based on a spreadsheet containing Article Reference No., Author, Project Name, Year, Country, Target (Patients or Group), Purpose of Technology Application for Personalized Care, Condition or Disease, living place (at Home or Elderly Care Center and etc.), Addressed Challenges or Barriers, Addressed Opportunity or Benefits which presented in Table A1 in Supplementary 1. Of the total reviewed publications, 23.8% have been published in 2019.

**Figure 2:**
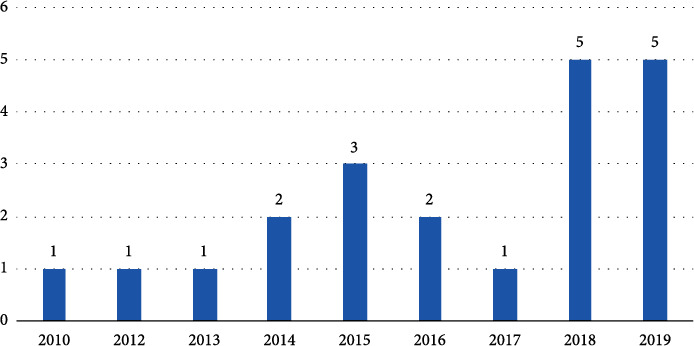
Articles published based on year, from 2010 to 2019.

From a total of 1895 search results, 129 studies were selected for the full-text review. Finally, 21 studies were included in the review according to the inclusion criteria (Figure 2). Most studies (about 5.21%, 5 articles) were published in 2019, and the fewest ones (1.21%) in 2012.

Studies had mentioned the challenges and benefits of different forms of living in the personalized home or elderly care center, e.g., at home or in residential long-term care facilities, primary care centers, hospitals, academic medical centers, clinics (community health centers), departments of veterans' affairs, and departments of cardiovascular care.

Studies had also addressed the challenges and benefits in different purposes of technology application for personalized care, e.g., self-management, supporting the elderly to live an independent life, individualized exercises, games, education, to help caregivers, friends, and family to avoid harm to the patients, personalized monitoring and rehabilitation services for older people, providing tele rehabilitation, and secondary stroke prevention. Furthermore, most efforts in personalized mobile technology applications for the elderly dealt with diabetes and chronic heart failure (CHF).

Numerous challenges and benefits related to the use of personalized mobile technology applications have been addressed, and a comprehensive classification of them has been prepared. Hudson and Cohen presented three important classified challenges for the use of personalized mobile technology applications: logistical, financial, and technical [37]. Poulsen mentioned the manner of implementation, controllability, Internet skills, and technology acceptance as the greatest challenges in this area [26]. Besides, Lee et al. expressed the greatest difficulties in using the monitor as operational problems and equipment quality [38].

Based on benefits and opportunities, Lefler et al. noted that increasing the quality of care, based on reducing the length of stay and the costs of m-Health equipment that can potentially improve patient-centered outcomes and self-management in older adults [39]. Based on the study by Li et al., usefulness, compatibility, facilitating conditions, and receiving immediate feedback on their physical conditions anywhere and anytime are the most important benefits of personalized mobile technology applications [40].

**Figure 3:**
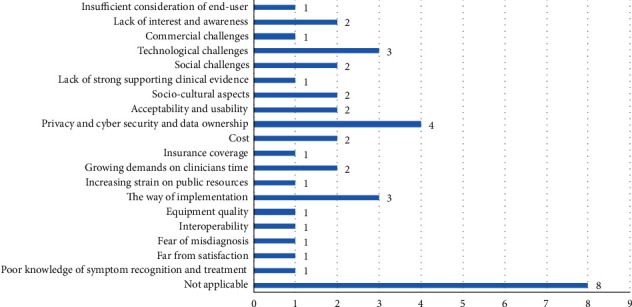
Frequency of addressed challenges of applying personalized mobile apps for elderly care.

The major benefits and challenges mentioned in the studies are given in Tables A2 and A3 in Supplementary 1. Based on Figure 3 and Table A2 in Supplementary 1, privacy and cybersecurity and data ownership are among the most mentioned challenges, while subjects that have received less attention are a lack of strong supporting clinical evidence, insurance coverage, the increasing strain on public resources, equipment quality, interoperability, and dissatisfactory and poor knowledge of symptom recognition and treatment.

Results indicated that 25% of the challenges were related to (1) privacy, cybersecurity, and data ownership (*N* = 4, 10%), (2) technology (*N* = 3, 7.5%), and (3) implementation (*N* = 3, 7.5%). The lack of strong supporting clinical evidence (*N* = 1, 2.5%), insurance coverage (*N* = 1, 2.5%), increasing strain on public resources (*N* = 1, 2.5%), equipment quality (*N* = 1, 2.5%), interoperability (*N* = 1, 2.5%), and dissatisfactory and poor knowledge of symptom recognition (*N* = 1, 2.5%) were the less frequently mentioned challenges. The most frequent opportunities were the benefits of the telecare system for cost-saving, nurse engagement improvement, and caregiver stress reduction.

**Figure 4:**
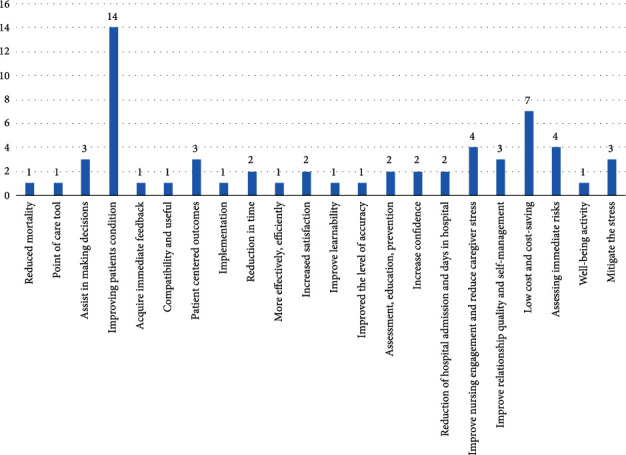
Frequency of addressed benefits of applying personalized mobile apps for elderly care.

According to Figure 4, low costs and cost savings, followed by improving health providers' engagement and reducing caregiver stress received more attention, in contrast to accuracy, learnability, and efficiency.

Based on findings, most elderly personalized mobile technologies were used for chronic conditions such as DM (diabetes mellitus), COPD (chronic obstructive pulmonary disease), dementia, and cardiovascular conditions (a total of 76%) (Table 2).

**Figure 5:**
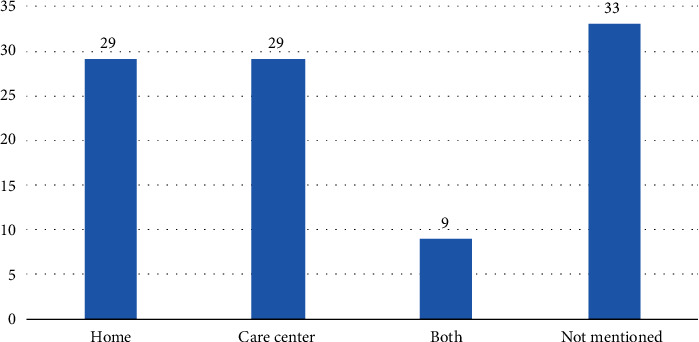
The places that elderly personalized mobile apps were used.

As can be seen in Figure 5, personalized mobile technology applications for the elderly have been developed for home use as much as for care centers.

## 4. Discussion

This review was conducted to investigate the challenges and benefits of applying PMH for the elderly. It is showed that most elderly personalized mobile technologies were developed in the USA and Europe (17 studies, 81%). Three studies were conducted in Asia, two of which were in China (10%). This might be due to the large elderly population in European countries and the high level of attention paid in these countries to them.

A growing population of the elderly will suffer several kind of diseases, especially chronic diseases, which is costly for societies [41]. According to the World Health Organization (WHO)'s report in 2008, 27 million people that died due to chronic diseases were over 60 years of age, which is about 75% of all deaths [42]. Health-related solutions increase self-care in chronic disease, improve clinical outcomes, and reduce costs [43]. Patients' desire for self-management and the development of mobile technology personalization programs depend heavily on patient control [44]. Furthermore, many older people want to spend time in their home environment [45]. Nearly 40% of the world's elderly population live independently due to their living conditions and chronic diseases. Thus, the use of PMH is useful for managing the chronic illnesses of older people [45, 46]. PMH and medical technologies can prevent dangerous situations with additional monitoring and rehabilitation services, as well as depression and social isolation improvement [46–48].

The Pew Foundation found that the percentage of older adults using technology, such as smartphones, the Internet, tablets, and social media, has increased steadily since 2000 [49]. PMH can allow elder people to stay in the home environment longer, resulting in reduced costs and improved quality of life [50]; despite all its advantages, this technology also has disadvantages.

Understanding the challenges in this area may lead to the establishment of a credible evaluation framework for reviewing PMH systems and solutions that are useful to project developers and managers because they can prevent future problems. In addition, this knowledge may be useful to policymakers nationally and internationally to make more effective use of information technology for health purposes by enacting effective and efficient policies, regulations, and guidelines.

The most important challenge is the challenge of all health information systems and all health stakeholders [51]. Unclear privacy, cybersecurity, and data ownership are critical issues that may endanger individuals' data if not given due consideration. The dilemmas around privacy and autonomy versus safety have already been reported, and their consideration is crucial for the acceptance of an eHealth service [37]. For many, concerns about privacy and exploitation outweighed the potential benefits [42]. Failure to meet this challenge may make the systems either rejected or used improperly.

Concerns about privacy and security may negatively impact patient and provider trust and adoption, pose a risk to mobile technology applications' success, and more broadly, to their expanded use. A study by Patel et al. indicated that privacy and security concerns are often noted as challenges to HIT adoption [39, 42]. However, patients are less concerned about privacy than providers and are generally more likely to use technology if they know the benefits outweigh the risks. Thus, although 33% of patients expressed privacy concerns, 64% of them reported a strong interest in using the proposed system [52].

In order to discuss and categorize the benefits and challenges of PHM, the result of this study is compared with a review conducted by Baniasadi et al. [53]. According to [53], there are six main categories of challenges for mobile-based systems for healthcare. Challenges in monitoring systems were indicated as follows: first, the user-related challenges which are mainly for users of system, composed of different levels of users' issues including digital literacy, lack of technology acceptance, lack of enough commitment to use technology, and weak communication between caregivers and patients. Second, the infrastructure challenges which are not under the control of the developer and related to standard, regulations (e.g., insurance coverage), and communication technologies such as Bluetooth, Wi-Fi, cellular data connection, and their technical features. Third, the process challenges which are not related to a specific part of the system and must be considered in each component of the system separately. In these challenges, several complications, including security (e.g., authentication and authorization), confidentiality, and ethical concerns, were considered. Fourth, management consisting of difficulties related to weak quality control and legislation. Fifth, the resource-related challenges which are corresponded to hardware, software, lack of specialized developers, and cost. Six, the training challenges related to those issues that are due to the lack of user training and instruction.

The results of the current review revealed that several of the challenges of [53] are repeated focusing on the pros and cons of PMH. The common challenges between these two studies were infrastructure, process, and resource-related ones. Having to look at these challenges in more details, presented in Figure 3, the infrastructure-related challenges of PMH are technological, privacy and cyber security, the way of system implementation, equipment quality, and interoperability. The process challenges are insufficient consideration of the end-user, social challenges, sociocultural aspects, lack of awareness and interest, poor knowledge of diagnosis and treatment, acceptability and usability, fear of misdiagnosis, and far from satisfaction. The resource-related challenges are cost, commercial challenges, lack of strong clinical evidence, insurance coverage, and increasing the time of clinicians. These three categories of challenges are more fundamental and require further actions for PMH's success. They could be researched to find procedures, amendment, solutions, high-technology applications such as blockchain, artificial intelligence, Internet of things, and cloud computing, and more resource allocation to promote PM. The latest solution can be conducted through PMH start-up companies' establishment to create added value from small investments to bigger resources.

Three of the challenges including user-related challenges, training, and management revealed in [53] become the benefits of PMH for the elderly, presented in Figure 4. According to the results of this study, for the category of management, there are benefits including assisting in decision-making, immediate risk assessment, low cost and cost-saving, nurse engagement improvement, reduce hospital admission and stay, higher level of accuracy, improve efficiency, time-saving, acquiring immediate feedback, better implementation, and lower mortality rate. The benefits of PMH for elderly users were stress mitigation, well-being activity, self-management improvement, confidence increase, improved satisfaction, and improved patient condition. For education and training advantages of personalized mobile applications for the elderly, there are improved learnability, better compatibility and usefulness, and improved education and prevention. A comparison of the results of these two studies showed that PMH is a further step of m-Health and an improvement in digital health which needs more development. This study shed light on those points that need to be highlighted for further work in order to convert the challenges toward benefits.

Paying attention to the user-friendly design of the information equipment can increase the acceptance of the technology. For example, because most older people are retired or unemployed and their chronic health conditions required extensive lifelong management, forcing users to pay for a long-term health plan creates an economic burden. However, according to surveys, most participants were willing to use the program if it was cost-effective [54].

Personalized mobile applications for the elderly can have significant effects on their quality of life, reduce mortality, improve patient experiences, and alleviate life stress associated with illness and depression. Most studies refer to the improvement of the patient's condition and reducing costs [36, 43, 46, 47, 54–61]. To improve the mobile applications to become more personalized, it is required to apply more intelligent features to the current version. That is, more predictive and detective features which work based on data mining and analytical techniques are required. Although there are applications developed for other purposes [62], more personalized mobile apps for elderly care are required. It is due to the fact that older groups have different needs for protecting their health and managing their illness; thus, it is required that e-health to be more intelligent and tailored based on patient's characteristics, and its benefits be promoted. Therefore, people must be aware of the potential benefits of using e-health [44].

## 5. Conclusions

Elderly caregiving is often hard and complex because of the heavy burden of their conditions. Given the increasing numbers of older people with chronic illnesses and the growing problems with clinical reasons, health systems must use technology-based solutions. This study was an overview of PMH implemented for the elderly and successfully established a list of benefits and challenges for elderly care. In general, the number of benefits in this study was slightly higher than the challenges; these findings related to barriers will help in realizing the challenges of developing information systems. Future research can focus on the issues of privacy and cybersecurity. Considering the benefits of using personalized mobile technology in the elderly, as well as trying to solve problems and barriers, this can make health organizations more effective in supporting modern care and achieving three goals of better care, better health, and lower costs. This support strengthens the community, health systems, self-management, service delivery systems, decision support systems, and clinical information systems.

## Figures and Tables

**Table 1:** PubMed search query.

(Wearable Electronic Devices[MeSH Terms] OR wearable device[TITLE /Abstract] OR Wearable Technolog^∗^ [TITLE /Abstract] OR Electronic Skin [TITLE /Abstract] OR telemedicine[MeSH Terms] OR telemedicine[TITLE /Abstract] OR mobile health[TITLE /Abstract] OR m-health[TITLE /Abstract] OR mhealth[TITLE /Abstract] OR telehealth[TITLE /Abstract] OR ehealth[TITLE /Abstract] OR Mobile Applications[MeSH Terms] OR app[TITLE /Abstract] OR apps[TITLE /Abstract] OR application^∗^[TITLE /Abstract] OR mobile device[TITLE /Abstract] OR Medical Informatics Applications[MeSH Terms] OR Smartphone[MeSH Terms] OR smartphone[TITLE /Abstract] OR Computers, Handheld[MeSH Terms] OR Handheld Computer [TITLE /Abstract] OR PDA[TITLE /Abstract] OR personal digital assist^∗^[TITLE /Abstract] OR computer palmtop[TITLE /Abstract] OR Palm-Top Computer[TITLE /Abstract] OR palm pilot[TITLE /Abstract] OR pocket pc[TITLE /Abstract] OR pocket personal computer[TITLE /Abstract] OR tablet [TITLE /Abstract])

AND (Self Care[MeSH Terms] OR Self Care[TITLE /Abstract] OR Self-Care[TITLE /Abstract] OR SelfCare[TITLE /Abstract] OR Self-Management[MeSH Terms] OR self-management[TITLE /Abstract] OR self-management [TITLE /Abstract] OR Home Nursing [MeSH Terms] OR Home Nursing [TITLE /Abstract] OR Home-Nursing [TITLE /Abstract] OR Home care [TITLE /Abstract] OR Home-care [TITLE /Abstract] OR Homecare[TITLE /Abstract] OR Home Care Services [MeSH Terms] OR Domiciliary Care [TITLE /Abstract] OR Home Health Aides[MeSH Terms] OR Home Aide [TITLE /Abstract] OR Home-Aide [TITLE /Abstract] OR Health Aide [TITLE /Abstract] OR Health-Aide [TITLE /Abstract] )

AND (Precision Medicine [MeSH Terms] OR precision medicine [ALL] OR personali^∗^ [ALL] OR Individuali^∗^ [ALL] OR p-health[ALL] )

AND (aged[MeSH Terms] OR aged[TITLE /Abstract] OR elderly[TITLE /Abstract] OR senility[TITLE /Abstract] OR old age [TITLE /Abstract] ) OR geriatric^∗^ [TITLE /Abstract]

**Table 2:** Frequency of studies based on the disease or condition.

Disease or condition	Count of studies	Percentage
Chronic diseases (diabetes, COPD, stroke, dementia, cardiovascular disease, hypertension)	16	76
Other conditions (improving the quality of life, fall prevention, controlling vital signs, increasing the quality of healthcare)	3	14
Not applicable	2	10

## Data Availability

The data used to support the findings of this study are included within the supplementary information file (Supplementary 2).

## Supplementary Materials

Supplementary 1 Three tables related to benefits and challenges extracted from the articles.

Supplementary 2 Excel file of complete information extracted from the articles.
