# Supplementary material for: Personalized Mobile Health for Elderly Home Care: A Systematic Review of Benefits and Challenges
Source: Int J Telemed Appl. 2023 Jan 16;2023:5390712. doi: 10.1155/2023/5390712 (PMC9871396; doi:10.1155/2023/5390712)
Supplement: Supplementary 1 — Three tables related to benefits and challenges extracted from the articles. [file 5390712.f1.docx]

Table A1. The most mentioned Benefits and Challenges of personalized mobile technology applications for elderly home care in reported by the reveiwed studies.

| **Article Reference Number** | **Author** | **Project** | **Year** | **Country** | **Target Patients/Group** | **Purpose of Technology Application for Personalized Care** | **Condition/ Disease** | **Living at Home or Elderly Care Center** | **Addressed Challenges** | **Addressed Benefits** |
| --- | --- | --- | --- | --- | --- | --- | --- | --- | --- | --- |
| 1 (47) | Adu MD, Malabu UH, Malau-Aduli AEO, Malau-Aduli BS | Users’ preferences and design recommendations to promote engagements with mobile apps for diabetes self-management: Multi-national perspectives | 2018 | 4 continents (Australia, Europe, Asia and America) | Adults, 18–76 years | Self-management (Blood pressure tracker, Blood glucose tracker, Food calorie counter, Fitness/exercise monitor, Body weight monitor, Transfer of health data to doctor, Reminder (e.g take medication, BG^a^ monitoring) and others | DM type1, 2 | NOT APPLICABLE | Insufficient:  -consideration of end-users  -perspectives and usability requirements. Lack of interest and awareness | -improve treatment  - mitigate stress |
| 2 (31) | Al-Shaqi R, Mourshed M, Rezgui Y | Progress in ambient-assisted systems for independent living by the elderly | 2016 | Wales, UK | Elderly | Support the elderly to live an independent life; help care givers, friends and family; and avoid harm to the patients | NOT APPLICABLE | Patients’ place of residence | -Commercial challenges  -Technological challenges  -Social challenges -lack of strong supporting clinical evidence  -Socio‑cultural aspects such as divergence among groups  -acceptability and usability  -privacy and cyber security | -aﬀecting wellbeing activities  -monitoring for assessing immediate risks  - low cost |
| 3 (43) | De San Miguel K, Smith J, Lewin G | Telehealth remote monitoring for community-dwelling older adults with chronic obstructive pulmonary disease | 2013 | Western Australia | 54 to 88 | Self-monitoring | COPD | Home | NOT APPLICABLE-It is also essential that we understand how long the telehealth monitoring service needs to be provided to be most cost effective. | -cost savings  -improve self-management  -provide measureable health benefits for people living with COPD  -reduce ED presentations, hospital admissions and hospital length of stay  - reduce health service contacts  -Participants reported benefits relating to increased confidence in self-management  -improved sense of security and reduced anxiety  -increased their personal awareness  -prompted more communication |
| 4 (32) | Delmastro F, Dolciotti C, La Rosa D, Di Martino F, Magrini M, Coscetti S, et al | Experimenting Mobile and e-Health Services with Frail MCI Older People | 2019 | Italy | Older people | Proposes a set of personalised monitoring and rehabilitation services for older people | Improving the quality of life | Home or residential long-term care facilities | NOT APPLICABLE | -lower the risk of depression and social  - prevent risky situations  -improve nursing engagement |
| 5 (48) | Dodakian L, McKenzie AL, Le V, See J, Pearson-Fuhrhop K, Burke Quinlan E, et al | A Home-Based Telerehabilitation Program for Patients With Stroke | 2017 | UC Irvine | 37 TO 71 | Providing telerehabilitation, education, and secondary stroke preventio  individualized exercises and games, stroke education, and an hour of free-play | Stroke | Home-based system | NOT APPLICABLE | -monitor and improve the health of patients after stroke  -use of games to drive treatment.  -assessment, education, prevention |
| 6 (49) | Gaugler JE, McCarron HR, Mitchell LL | Perceptions of precision medicine among diverse dementia caregivers and professional providers | 2019 | Minneapolis, MN | Mean age of caregivers and health professionals: 50 | To understand how different family caregivers and health-care professionals view the benefits and risks of precision medicine as well as cultural dimensions to consider when developing and implementing precision medicine interventions in dementia care | Dementia | NOT APPLICABLE | -Concerns included cost of precision medicine and insurance coverage  -lack of alignment of PM with cultural norms and values  -unclear privacy and data ownership | - prevent dementia  - help families prepare for a diagnosis  - reduce caregiver stress |
| 7 (33) | Hamm J, Money AG, Atwal A | Enabling older adults to carry out paperless falls-risk self-assessments using guidetomeasure-3D: A mixed methods study | 2019 | UK | Older adult patients | Fall prevention intervention strategy | Fall prevention intervention strategy | Home environment | Increased older adult patient autonomy promises to deliver crucial efficiencies which are much needed, given the growing demands on clinicians’ time and the increasing strain on public resources. | -improved level of accuracy using  - increased satisfaction  -increased confidence levels.  -Improved learnability  -more effectiveness and efficiency  -improved levels of service user  -improved overall patient satisfaction, quality of life  - fall prevention. |
| 8 (37) | Hudson DL, Cohen ME | Intelligent agents in home healthcare | 2010 | San Francisco, CA, USA | Elderly | Remote monitoring | Cardiac-related problems and+ diminishing mental capacity | Home healthcare | Many obstacles:  -logistic  - financial  - technical | Reducing costs  - supporting family members  -helping remote care provider  -identifying potential problems  -improves quality of life.  -Very reduction in time and travel |
| 9 (38) | Huygens MWJ, Vermeulen J, Swinkels ICS, Friele RD, Van Schayck OCP, De Witte LP | Expectations and needs of patients with a chronic disease toward self-management and ehealth for self-management purposes | 2016 | The Netherlands | Range: 50–83 | Self-management; To investigate expectations and needs of people with a chronic condition regarding self-management and eHealth for self-management purposes, their willingness to use ehealth, and possible differences between patient groups | Chronic condition (diabetes, COPD, cardiovascular disease) | Primary care centres | -aspects of eHealth, and the manner of implementation -controllability  -Internet skills  -technology acceptance | Implementation of eHealth with budget cuts in care |
| 10 (39) | Lee TT, Huang TY, Chang CP, Lin KC, Tu HM, Fan CJ, et al | The evaluation of diabetic patients' use of a telehealth program | 2014 | Taiwan | Mean: 59 | Ultimate goal of the telehealth program was to encourage patients with chronic diseases to adopt a proactive behavior, actively monitor their health, and receive medical supervision at all times, thereby enhancing the healthcare behaviors of the patients. | Diabetes | Hospital telecare platform and the outpatient monthly reporting system | The greatest difficulties in using the monitor were operational problems and equipment quality | -using the telehealth monitor effectively  -improved diet control, blood glucose levels, blood pressure values, and hba1c values |
| 11 (40) | Lefler LL, Rhoads SJ, Harris M, Funderburg AE, Lubin SA, Martel ID, et al | Evaluating the use of mobile health technology in older adults with heart failure: Mixed-methods study | 2018 | United States | Aged 55 years and above | Improving self-care and HF management | Heart failure | Home | NOT APPLICABLE | Increasing the quality of care while reducing hospitalizations and costs of mHealth equipment is feasible with the potential to improve Personalized outcomes and increase self-management in older adults with HF |
| 12 (41) | Li J, Ma Q, Chan AH, Man SS | Health monitoring through wearable technologies for older adults: Smart wearables acceptance model | 2019 | Hong Kong | 60 years and above | Vital physical signs measured by smart wearable systems | Vital physical signs | NOT APPLICABLE | NOT APPLICABLE | -usefulness  -compatibility  -facilitating conditions  -receiving immediate feedback on their physical conditions anywhere and anytime |
| 13 (34) | Nundy S, Dick JJ, Chou CH, Nocon RS, Chin MH, Peek ME | Mobile phone diabetes project led to improved glycemic control and net savings for Chicago plan participants | 2014 | Chicago | 18 years or older | The program is a theory-driven behavioral intervention designed to improve self-care through multiple mediators, including cuing, education, self-efficacy, social support, and health beliefs. | Type 1 or type 2 diabetes | In a healthcare setting (an academic medical center) | -sustainability of mHealth  -accountability  - interoperability | -improving patients’ experiences  -improving population health, and reducing the per capita cost  - improving clinical outcomes -better healthcare  -lower costs |
| 14 (50) | Patel V, Hale TM, Palakodeti S, Kvedar JC, Jethwani K | Prescription Tablets in the Digital Age: A Cross-Sectional Study Exploring Patient and Physician Attitudes Toward the Use of Tablets for Clinic-Based Personalized Healthcare Information Exchange | 2015 | Massachusetts General Hospital (MGH) in Boston | Mean age: 43 | The aim of this study is to explore patient and provider attitudes and interest in a proposed clinic-based tablet system for personal health information exchange. | NOT APPLICABLE | Clinic (a community health center) | -security of data  - feasibility of using tablets in the clinic.  -difficulty using tablets | -improve clinical workflow and patient education - improv patients’ health knowledge and patient reported outcome measures  -improve patient-provider communication. -assist in making decisions  -Engage patients in their health and medical care  - reduce the time  A point of care tool. |
| 15 (51) | Pérez-Gandía C, García-Sáez G, Subías D, Rodríguez-Herrero A, Gómez EJ, Rigla M, et al | Decision Support in Diabetes Care: The Challenge of Supporting Patients in Their Daily Living Using a Mobile Glucose Predictor | 2018 | Spain | Average age of 41.97 ± 9.30 years | Own care  telemedicine platform allowed participants to register monitoring data and decisions and allowed endocrinologists to supervise data at the hospital. | Type 1 diabetes mellitus (T1DM) | NOT APPLICABLE | NOT APPLICABLE | Patients’ decision-making |
| 16 (52) | Piette JD, Striplin D, Marinec N, Chen J, Trivedi RB, Aron DC, et al | A mobile health intervention supporting heart failure patients and their informal caregivers: A randomized comparative effectiveness trial | 2015 | The United States | Participants were on average 67.8 years of age. | Self-care | Heart failure (HF) | We identified 331 HF patients from the Department of Veterans' Affairs outpatient clinics. All patients identified a “carepartner” outside their household. | NOT APPLICABLE | -providing caregivers with automated updates and guidance on self-care  -decrease patients’ risk of HF  - improve quality of life among patients with greater depressive symptoms.  -improve relationship quality and self-management |
| 17 (53) | Rasche P, Wille M, Bröhl C, Theis S, Schäfer K, Knobe M, et al | Prevalence of health app use among older adults in germany: National survey | 2018 | Germany | Older adults | Self-management | To increase quality in healthcare and thus the QoL of patients  Participants suffered on average from at least one chronic disease, whereby the most frequently reported diseases for all three groups were hypertension and back pain. | NOT APPLICABLE | -lack of trust, data privacy concerns, and fear of misdiagnosis | NOT APPLICABLE |
| 18 (54) | Schaller S, Marinova-Schmidt V, Gobin J, Criegee-Rieck M, Griebel L, Engel S, et al | Tailored e-Health services for the dementia care setting: A pilot study of 'ehealthmonitor' | 2015 | Germany | Mps mean age was 43 years ( min = 25; max = 58)  caregivers mean age was 58 years, (25 to 83 years old) | To provide targeted and personalized support for informal caregivers of people with dementia | Dementia | A home-based care setting | -the implementation of a chatroom for caregivers, an upload function, and alerts | -emphasize the potential of personalized and web-based support services for caregivers  -Assistance in decision-making  -empowering caregivers  -reducing costs -acquisition of individualized information  -computerized interaction between caregivers and medical professionals |
| 19 (55) | Sohn S, Helms TM, Pelleter JT, Müller A, Kröttinger AI, Schöffski O | Costs and benefits of personalized healthcare for patients with chronic heart failure in the care and education program "telemedicine for the heart" | 2012 | Germany | Mean age: 65 | Self-measurements | Heart failure | NOT APPLICABLE | NOT APPLICABLE | -reduction of costs  - reduced hospital length of stay  -optimized medical therapy  -better quality of life  - reduced mortality |
| 20 (56) | Still CH, Jones LM, Moss KO, Variath M, Wright KD | African American older adults’ perceived use of technology for hypertension self-management | 2018 | African-American | Older adults- from 62 to 91 years, with a mean age of 72 | Self-management | Hypertension | NOT APPLICABLE | May help improve blood pressure control and address important clinical and public health priorities of uncontrolled hypertension | -Hypertension self-management practices  -adherence to antihypertensive drugs, and lifestyle  -improved blood pressure control and better cardiovascular outcomes |
| 21 (57) | Sun J, Zhang ZW, Ma YX, Liu W, Wang CY | Application of self-care based on full-course individualized health education in patients with chronic heart failure and its influencing factors | 2019 | China | Age group 68.21 ± 4.69 Control group 68.57 ± 4.12 | Self-care | Patients with chronic heart failure (CHF) | Department of Cardiovascular Diseases | -far from satisfactory.  -Poor knowledge of symptom recognition and treatment and lack of confidence in treatment have restricted the implementation of self-care. | Improved:  - self-care behavior and cardiac function in CHF patients.  -education level -achieve therapeutic effects |

**Table A2.** Recognized challenges by repetition per study and year.

| **Row** | **Challenge type** | **Article Number in Table A1** | **Study year** |
| --- | --- | --- | --- |
| 1 | Insufficient consideration of end-users | 1, | 2018 |
| 2 | lack of interest and awareness | 1, 14 | 2018, 2015 |
| 3 | Commercial challenges | 2 | 2016 |
| 4 | Technological challenges | 2,8,9 | 2016, 2010, 2016 |
| 5 | Social challenges | 2,13 | 2016, 2014 |
| 6 | lack of strong supporting clinical evidence | 2 | 2016 |
| 7 | Socio‑cultural aspects | 2,6 | 2016, 2019 |
| 8 | Acceptability and usability | 2,9 | 2016, 2016 |
| 9 | Privacy, cyber security, and data ownership | 2,6,14,17 | 2016, 2019.2015, 2018 |
| 10 | Costs | 3,6 | 2013, 2019 |
| 11 | Insurance coverage | 6 | 2019 |
| 12 | Growing demands on clinicians’ time | 7,10 | 2019, 2014 |
| 13 | Increasing strain on public resources | 7 | 2019 |
| 14 | The manner of implementation | 9,18,21 | 2016, 2015, 2019 |
| 15 | Equipment quality | 10 | 2014 |
| 16 | Interoperability | 13 | 2014 |
| 17 | Fear of misdiagnosis | 17 | 2018 |
| 18 | Far from satisfactory | 21 | 2019 |
| 19 | Poor knowledge of symptom recognition and treatment | 21 | 2019 |
| 20 | NOT APPLICABLE | 4,5,11,12,15,16,19,20 | 2019, 2017, 2018, 2019, 2018, 2015, 2012, 2018 |

**Table A3.** Recognized benefits by repetition per study and year.

| **Row** | **benefit type** | **Article Number in table A1** | **Study year** |
| --- | --- | --- | --- |
| 1 | Mitigating stress | 6, 1, 3 | 2019, 2018, 2013 |
| 2 | Well-being activities | 2 | 2016 |
| 3 | Assessing immediate risks | 4, 6, 7, 16 | 2019, 2019, 2019, 2015 |
| 4 | Low costs and cost savings | 2, 3, 8, 11, 13, 18, 19 | 2016, 2013, 2010, 2018, 2014, 2015, 2012 |
| 5 | Improving relationship quality and self-management | 3, 11, 16 | 2013, 2018, 2015 |
| 6 | Improving nursing engagement and reducing caregiver stress | 4, 6, 16, 18 | 2019, 2019, 2015, 2015, |
| 7 | Reducing hospital admissions and length of stay | 3, 19 | 2013, 2012 |
| 8 | Increasing confidence | 3, 7 | 2013, 2019 |
| 9 | Assessment, education, prevention | 14, 21 | 2015, 2019 |
| 10 | Improving the level of accuracy | 7 | 2019 |
| 11 | Improving learnability | 7 | 2019 |
| 12 | Increasing satisfaction | 7, 21 | 2019, 2019 |
| 13 | More effectiveness and efficiency | 7 | 2019 |
| 14 | Reducing time | 8, 12 | 2010, 2019 |
| 15 | Implementation | 9 | 2016 |
| 16 | Personalized outcomes | 13, 14, 20 | 2014, 2015, 2018 |
| 17 | Compatibility and usefulness | 12 | 2019 |
| 18 | Receiving immediate feedback | 12 | 2019 |
| 19 | Improving patients’ condition | 1, 3, 4, 5, 7, 8, 10, 11, 12, 13, 14, 16, 20, 21 | 2018, 2013, 2019, 2017, 2019, 2010, 2014, 2018, 2019, 2014, 2015, 2015, 2018, 2019 |
| 20 | Assisting in making decisions | 14, 15, 18 | 2015, 2018, 2015 |
| 21 | Point of care tool | 14 | 2015 |
| 22 | Reducing mortality | 19 | 2012 |
| 23 | NOT APPLICABLE | 17 | 2018 |
