# Supplementary material for: Personalized Mobile Health for Elderly Home Care: A Systematic Review of Benefits and Challenges
Source: Int J Telemed Appl. 2023 Jan 16;2023:5390712. doi: 10.1155/2023/5390712 (PMC9871396; doi:10.1155/2023/5390712)
Supplement: Supplementary 2 — Excel file of complete information extracted from the articles. [file 5390712.f2.pdf]

Addressed Opportunity / Benefits	Addressed Challenges / Barriers	Living Home or Elderly Care Center	Condition/ Disease	Purpose of Technology Application for Personalized Care	Target Patients/Group	Country/	Year	Project	auters	Article Reference No
improve treatment adherence by encouraging optimism, which can mitigate the stress of living with the illness and accompanying depression,	insufficient consideration of end-users perspectives and usability requirements.<br>lack of interest and awareness(apps are ambiguous,and not provide clear information on the specific health benefits for patients),Do not have a smart phone,Lack of access to the internet,Expensive,Missing. redundancy (if consolidation of data from peripheral devices into app).<br>participants proposed future apps that are user friendly, support healthy eating, provide actionable reminders and consolidate data across peripheral health devices. customised features with news update on developments in the field of diabetes. Nominated specific educational topics included tips on problem solving, use of insulin pump therapy, smore comprehensive diet management features in future apps, comprising of nutrient data base of both the common and rare foods, and recipes for tasty foods which are suitable for people with diabetes.more comprehensive diet management features in future apps, comprising of nutrient data base of both the common and rare foods, and recipes for tasty foods which are suitable for people with diabetes	NOT APPLICABLE	DM type1, 2	self-management (Blood pressure tracker,Blood glucose tracker,Food calorie counter,Fitness/exercise monitor,Body weight monitor,Transfer of health data to doctor,Reminder (e.g take medication, BGa monitoring) and others	adults- 18–76 years	4 continents (Australia, Europe, Asia and America)	2018	Users' preferences and design recommendations to promote engagements with mobile apps for diabetes self-management: Multi-national perspectives	Adu MD, Malabu UH, Malau-Aduli AEO, Malau-Aduli BS	1
affecting their wellbeing,activity monitoring for assessing immediate risks, low cost	Commercial challenges,Technological challenges,Social challenges<br>(The potential for wearable devices and sensors, as well as distributed storage and access (e.g. cloud) are yet to be fully appreciated.<br>There is a distinct lack of strong supporting clinical evidence from the implemented technologies.<br>Socio-cultural aspects such as divergence among groups, acceptability and usability of AALS were also overlooked. Future systems need to look into the issues of privacy and cyber security)	patients' place of residence	NOT APPLICABLE	supporting the elderly to live an independent life; help care givers, friends and family; and to avoid harm to the patients	elderly	Wales, UK	2016	Progress in ambient assisted systems for independent living by the elderly	Al-Shaqi R, Mourshed M, Rezgui Y	2
significant costs savings,improvements in participants' self-management and control over their condition was evident and importantly, older people were receptive to using this type of technology and enjoyed using it as a tool in managing their COPD, provide measureable health benefits for people living with COPD,reduction in ED presentations, hospital admissions and days in hospital<br>provide users of the service with more than just reduced health service contacts. Participants reported benefits relating to increased confidence in self-management, control and awareness in managing their condition, improved sense of security and reduced anxiety, increased their personal awareness of their health status,prompted more communication about their condition with their GP and in some cases the monitoring results were used to open discussion with their GP about reviewing their medications,demonstrating that participants were taking a more proactive role in managing their condition	NOT APPLICABLE-It is also essential that we understand how long the telehealth monitoring service needs to be provided to be most cost effective.	home	COPD	self-monitoring	54 to 88	, Western Australia	2013	Telehealth remote monitoring for community-dwelling older adults with chronic obstructive pulmonary disease	De San Miguel K, Smith J, Lewin G	3
can lower the risk of depression and social , to prevent risky situations, including cognitive and motor decline, depression and social isolation, improve nursing engagement	NOT APPLICABLE	home or in residential long-term care facilities	Improving the quality of life	proposes a set of personalised monitoring and rehabilitation services for older people,	older people,	Italy	2019	Experimenting Mobile and e-Health Services with Frail MCI Older People dagger	Delmastro F, Dolciotti C, La Rosa D, Di Martino F, Magrini M, Coscetti S, et al	4

Addressed Opportunity / Benefits	Addressed Challenges / Barriers	Living Home or Elderly Care Center	Condition/ Disease	Purpose of Technology Application for Personalized Care	Target Patients/Group	Country/	Year	Project	auters	Article Reference No
monitor and improve the health of patients after stroke, use of games to drive treatment. patients were asked to sign a behavioral contract, which increases adherence, engagement, and accountability<br>patients could pause the system as needed between assigned tasks, ystem emphasized variety, with the different modules (therapy, assessment, education, prevention, videoconferencing), numerous games and exercises, and range of different hardware input devices incorporated into individual treatment plans<br>patients could phone the lab if a technical question arose<br>focused on ease of use, including simple, large-font instructions<br>effective system use did not require computer skills<br><br>to improve blood pressure control in patients with chronic stroke<br>effectively deliver telerehabilitation, improve patient education, screen for complications of stroke, and provide patients at home with a means for interaction with medical personnel<br>feasibility and potential utility of this home-based program for improving outcomes after stroke<br>(1) patients were highly compliant (97.9% of assigned days) and rated the system favorably; (2) therapists were readily able to review patient performance and revise therapy; (3) videoconferences supported regular communication between the patient and treatment team; (4) arm motor status improved significantly overall and exceeded the MCID in half of the subjects; (5) daily education increased stroke prevention knowledge by 39%; (6) screening for depression was accurate; (7) BP measurement was very accurate; and (8) all of these findings were unrelated to subjects' computer skills	NOT APPLICABLE	home-based system	stroke	providing telerehabilitation, education, and secondary stroke preventio individualized exercises and games, stroke education, and an hour of free-play	37 TO 71	UC Irvine	2017	A Home-Based Telerehabilitation Program for Patients With Stroke	Dodakian L, McKenzie AL, Le V, See J, Pearson-Fuhrhop K, Burke Quinlan E, et al	5
to prevent dementia, help families prepare for a diagnosis, and reduce caregiver stress, there is skepticism and concern for the use of PM in these communities.	Concerns included cost of precision medicine and insurance coverage; lack of alignment of PM with cultural norms and values; historically and currently fraught relationships between communities, health professionals, and researchers; unclear privacy and data ownership; and the potential trade-off of knowing risk versus treatment benefit were among the most notable concerns of our diverse sample of participants<br>many forms of PM are not integrated with the needs and concerns of underserved communities	NOT APPLICABLE	dementia	to understand how diverse family caregivers and health-care professionals view the benefits and risks of precision medicine as well as cultural dimensions to consider when developing and implementing precision medicine interventions in dementia care	age mean caregivers and health professionals 50	Minneapolis, MN	2019	Perceptions of precision medicine among diverse dementia caregivers and professional providers	Gaugler JE, McCarron HR, Mitchell LL	6
older adults have improved the level of accuracy using guidetomeasure-3D And efficiency along with increased satisfaction and increased confidence levels compared to the equivalent of Article 2D.<br>Improve Learnability. to empower older adult users to carry out assessments more effectively, efciently, and with enhanced levels of confdence and<br>improved levels of service user autonomy, hence enabling the provision of more personalised care.<br>improve overall patient satisfaction, quality of life, and ultimately, the level of engagement with assistive equipment for falls prevention.	Increased older adult patient autonomy promises to deliver crucial efciencies which are much needed, given the growing demands on clinicians' time and the increasing strain on public resources.	home environment	falls prevention intervention strategy	falls prevention intervention strategy	older adult patients	UK	2019	Enabling older adults to carry out paperless falls-risk self-assessments using guidetomeasure-3D: A mixed methods study	Hamm J, Money AG, Atwal A	7

Addressed Opportunity / Benefits	Addressed Challenges / Barriers	Living Home or Elderly Care Center	Condition/ Disease	Purpose of Technology Application for Personalized Care	Target Patients/Group	Country/	Year	Project	auters	Article Reference No
With remote interventions, the patient can remain at home, not only reducing costs but also benefiting from a familiar environment and support of family members help the remote care provider have a deeper picture of the patient's health, Remote access can help identify potential problems and can be used to communicate with family members.<br>These methods allow patients to live longer in the home environment, which reduces costs and improves quality of life.<br>very beneficial to the patient and the patient's family and also to the healthcare provider in reduction in time and travel	Many obstacles remain in completing the implementation of these methods including logistical, financial, and technical issues.	home healthcare	cardiac-related problems and diminishing mental capacity.	remote monitoring	elderly	San Francisco, CA, USA	2010	Intelligent agents in home healthcare	Hudson DL, Cohen ME	8
implementation of eHealth with budget cuts in care	aspects of eHealth, and the way in which it should be implemented, should be tailored to the patient.<br>Differences are found in expectations and needs between different patient groups regarding self-management and eHealth for self-management purposes, suggesting that eHealth and its implementation should be tailored to the patient group.<br>Patients' expected benefits of eHealth and their perceived controllability over their disease seem to be important in patients' willingness to use eHealth for self-management purposes. Informing patients clearly in a well-considered way about the possibilities, usage and reasons for implementation are important for stimulating the uptake of eHealth in primary care. However, when offering eHealth to patients it should be taken into account that not every patient is willing to use it.<br>degree of computer and Internet skills has an influence on technology acceptance.	primary care centres	chronic condition(diabetes, COPD, cardiovascular condition)	Self-management.<br>The objectives of this study are to investigate expectations and needs of people with a chronic condition regarding self-management and eHealth for self-management purposes, their willingness to use eHealth, and possible differences between patient groups regarding these topics.	range 50–83	Netherlands	2016	Expectations and needs of patients with a chronic disease toward self-management and eHealth for self-management purposes	Huygens MWJ, Vermeulen J, Swinkels ICS, Friele RD, Van Schayck OCP, De Witte LP	9
using the telehealth monitor effectively improved their diet control, blood glucose levels, blood pressure values, and HbA1c values	The greatest difficulties in using the monitor were operational problems and equipment quality	hospital telecare platform and the outpatientmonthlyreportingsystem	diabetes	ultimate goal of the telehealth program was to encourage patients with chronic diseases to adopt a proactive behavior, actively monitor their health, and receive medical supervision at all times, thereby enhancing the healthcare behaviors of the patients	mean 59	Taiwan	2014	The evaluation of diabetic patients' use of a telehealth program	Lee TT, Huang TY, Chang CP, Lin KC, Tu HM, Fan CJ, et al	10
increasing quality of care while reducing hospitalizations and costs<br>mHealth equipment is feasible with potential to improve patient-centered outcomes and increase self-management in older adults with HF	?	home	Heart failure	improving self-care and HF management	aged 55 years and above	United States	2018	Evaluating the use of mobile health technology in older adults with heart failure: Mixed-methods study	Lefler LL, Rhoads SJ, Harris M, Funderburg AE, Lubin SA, Martel ID, et al	11

Addressed Opportunity / Benefits	Addressed Challenges / Barriers	Living Home or Elderly Care Center	Condition/ Disease	Purpose of Technology Application for Personalized Care	Target Patients/Group	Country/	Year	Project	auters	Article Reference No
usefulness, compatibility, facilitating conditions, and selfreported health status significantly and positively affect older adults' intention to use such technologies enable users to acquire immediate feedback on their physical conditions anywhere and anytime.	?	NOT APPLICABLE	Vital physical signs	Vital physical signs measured by smart wearable systems	60 years and above	Hong Kong	2019	Health monitoring through wearable technologies for older adults: Smart wearables acceptance model	Li J, Ma Q, Chan AH, Man SS	12
mHealth programs can support health care organizations' pursuit of the triple aim of improving patients' experiences with care, improving population health, and reducing the per capita cost of health care<br>connected health solutions hold promise for supporting chronic disease selfcare, improving clinical outcomes<br>Our study offers early evidence that mHealth can enable health care organizations to effectively support patients beyond the traditional health care setting and achieve the triple aim of better health, better health care, and lower costs.	Although we found a business case for the use of mHealth, the diffusion and sustainability of mHealth depends on a supportive policy environment. Accelerated movement toward accountability for population health, increased interoperability with electronic health records, and clearer regulatory guidance will be important for unlocking mHealth's potential to support behavior change and chronic care.	in a health care setting(an academic medical center's)	type 1 or type 2 diabetes	The program is a theory-driven behavioral intervention designed to improve self-care through multiple mediators, including cuing, education, self-efficacy, social support, and health beliefs.	eighteen or older	Chicago	2014	Mobile phone diabetes project led to improved glycemic control and net savings for chicago plan participants	Nundy S, Dick JJ, Chou CH, Nocon RS, Chin MH, Peek ME	13
improve clinical workflow and patient education<br>Both patients and providers were highly amenable to integrating tablets into the clinical experience, and tablets may be useful in improving patients' health knowledge, the collection of patient reported outcome measures, and improved patient-provider communication.<br>A majority of patients believed the tablet system would have positive effects on their health knowledge, assist in making decisions regarding their health, and help them to feel more comfortable communicating with their provider. Patients thought the proposed system would be more engaging, motivating, and informative than other communication channels. Providers thought the tablet system would enable more personalized delivery of health education content to patients and the collection of patient-reported data for use during the clinical encounter<br>Engaging patients in their health and medical care is understood to be key to achieving better health outcomes and higher patient satisfaction with care .<br>Tablets used in an inpatient setting have been found to reduce the time required to check the EHR and increase the time providers spend with patients<br>A point of care tool that delivers information, prompts patients to take action or change behaviors, and supports patient-provider communication and shared decision making may be the most effective model to improving engagement.<br><br>Patients responded positively to the proposed tablet system and thought it would help them to improve their knowledge, assist in making decisions about their health, and feel more comfortable communicating with their providers.	Patients and providers were concerned about privacy and security of data collected using the tablets.<br><br>The primary barrier cited by physicians that limit the use of educational materials and patient-provider discussions on treatment decisions is time constraint.<br><br>Providers expressed some concerns about the feasibility of using tablets in the clinic. One concern was coordinating the distribution and return of equipment. A second concern of providers was that some patients may not be able to make effective use of tablet-based systems. Research has found that patients who are older and with lower levels of income and education have more difficulty using tablets	clinic (a community health center)	NOT APPLICABLE	The aim of this study is to explore patient and provider attitudes and interest in a proposed clinic-based tablet system for personal health information exchange	mean age 43	Massachusetts General Hospital (MGH) in Boston	2015	Prescription Tablets in the Digital Age: A Cross-Sectional Study Exploring Patient and Physician Attitudes Toward the Use of Tablets for Clinic-Based Personalized Health Care Information Exchange	Patel V, Hale TM, Palakodeti S, Kvedar JC, Jethwani K	14
The DSS had a relevant impact on patients' decision making while dealing with type 1 diabetes.<br>Patients' confidence in he glucose prediction was high, and they had a positive subjective assessment of the tool.	NOT APPLICABLE	NOT APPLICABLE	type 1 diabetes mellitus (T1DM)	own care telemedicine platform allowed participants to register monitoring data and decisions and allowed endocrinologists to supervise data at the hospital.	average age of 41.97 ± 9.30 years	Spain	2018	Decision Support in Diabetes Care: The Challenge of Supporting Patients in Their Daily Living Using a Mobile Glucose Predictor	Pérez-Gandía C, García-Sáez G, Subías D, Rodríguez-Herrero A, Gómez EJ, Rigla M, et al	15

Addressed Opportunity / Benefits	Addressed Challenges / Barriers	Living Home or Elderly Care Center	Condition/ Disease	Purpose of Technology Application for Personalized Care	Target Patients/Group	Country/	Year	Project	auters	Article Reference No
providing caregivers with automated updates and guidance on self- care support may enhance the beneficial effects of mHealth for HF patients'health and self-management<br>mHealth+CP may also decrease patients' risk of HF exacerbations related to shortness of breath and sudden weight gains. mHealth+CP may improve quality of life among patients with greater depressive symptoms.<br>this suggests that involvement in mHealth information exchange may significantly improve relationship quality and self-management assistance for patients with chronic health problems.	NOT APPLICABLE	We identified 331 HF patients from Department of Veterans Affairs outpatient clinics. All patients identified a "CarePartner" outside their household.	heart failure (HF)	self-care	Participants were on average 67.8 years of age	United States	2015	A mobile health intervention supporting heart failure patients and their informal caregivers: A randomized comparative effectiveness trial	Piette JD, Striplin D, Marinec N, Chen J, Trivedi RB, Aron DC, et al	16
NOT APPLICABLE	a lack of trust, data privacy concerns, and fear of misdiagnosis.	NOT APPLICABLE	to increase quality in health care and thus the QoL of patients Participants suffered on average from at least one chronic disease, whereby the most frequently reported diseases for all three groups were hypertension and back pain	self-management	older adults	Ghermany	2018	Prevalence of health app use among older adults in germany: National survey	Rasche P, Wille M, Bröhl C, Theis S, Schäfer K, Knobe M, et al	17
The perceived benefits and willingness to use the system, combined with an increasing number of adults who use the internet regularly, emphasize the potential of personalized and web-based support services for caregivers. Assistance in decision making and empowering caregivers are essential to lowering and preventing caregiver burden affiliated work and/or stresses. E-health interventions can be an efficient alternative to provide personalized support for caregivers at reduced costs<br>Upon implementing the reported pilot results into the eHM-DP, further research will focus on a) the cost-effectiveness and benefits of the eHM-DP in real-life settings (at home/at work), b) the perceived benefits for specific user subgroups as well as c) the integration of the eHM-DP into existing healthcare infrastructures<br>Caregivers indicated a high degree of perceived support by the eHM-DP and of provided decision aid.<br>The primary benefits participants perceived were the acquisition of individualized information, computerized interaction between caregivers and MPs, empowerment in health-related decisions and comprehensive insights into the progress of the disease<br>for caregivers with regard to caregiver empowerment (knowledge, decision aid), facilitated access to health care services, and promoting interaction with MPs	Major recommendations for improving the eHM-DP encompassed: an active search functionality based on predefined terms, the implementation of a chatroom for caregivers, an upload function and alerts for MPs, as well as the overall design.	in a home-based care setting	dementia	It aims to provide targeted and personalized support for informal caregivers of people with dementia	MPs mean age was 43 years (min = 25; max = 58) caregivers mean age was 58 years,(25 to 83 years old)	Germany	2015	Tailored e-Health services for the dementia care setting: A pilot study of 'eHealthMonitor'	Schaller S, Marinova-Schmidt V, Gobin J, Criegee-Rieck M, Griebel L, Engel S, et al	18

Addressed Opportunity / Benefits	Addressed Challenges / Barriers	Living Home or Elderly Care Center	Condition/ Disease	Purpose of Technology Application for Personalized Care	Target Patients/Group	Country/	Year	Project	auters	Article Reference No
The findings suggest that, besides a reduction of costs, by participating in ‘‘Telemedicine for the Heart’’ patients with chronic heart failure experienced a reduced number of hospital stays, optimized medical therapy, better quality of life, and reduced mortality	NOT APPLICABLE	NOT APPLICABLE	heart failure	selfmeasurements	age mean 65	German	2012	Costs and benefits of personalized healthcare for patients with chronic heart failure in the care and education program "telemedicine for the heart"	Sohn S, Helms TM, Pelleter JT, Müller A, Kröttinger AI, Schöffski O	19
Hypertension self-management practices, adherence to antihypertensive drugs, and lifestyle modification have been shown to be associated with improved blood pressure control and better cardiovascular outcomes.	Participants expressed concerns about not being informed or trained sufficiently to integrate technology for hypertension selfmanagement. There is a need to develop novel hypertension self-management interventions that integrate technology and training programs for this marginalized population that may help improve blood pressure control and address important clinical and public health priorities of uncontrolled hypertension	NOT APPLICABLE	hypertension	self-management	older adults-from 62 to 91 years, with a mean age of 72	African-American	2018	African American older adults' perceived use of technology for hypertension self-management	Still CH, Jones LM, Moss KO, Variath M, Wright KD	20
full-course individualized health education (FCIHE) helps to improve cardiac function in CHF patients, helps to improve self-care behavior, helps to improve quality of life,increasing their confidence in disease treatment.<br>improved self-care behavior and cardiac function in CHF patients. Age, cardiac function, and education level affected the implementation of self-care among CHF patients<br>self-care based on full-course individualized health education can better assist clinical treatment and can achieve therapeutic effects similar to treatment by intervening in the overall behavior of patients.<br>The individualization and continuity of health education was guaranteed, which, to a certain extent, ensured the seamless transition from hospital to family.<br>FCIHE has the following advantages. (1) The full-course dynamic management helps the medical staff to learn the patient's medical condition and self-care needs in a more comprehensive and real-time manner. According to the real situation of the patients, the health guidance programs can be adjusted timeously; (2) the full-course participation of medical staff in the health education of patients helps to establish a good doctor–patient relationship and thus increase adherence to treatment and care protocols; and (3) participation of multidisciplinary teams can facilitate healthcare professional development and increase their ability to serve patients.	Currently, self-care among CHF patients is far from satisfactory. Poor knowledge of symptom recognition and treatment and lack of confidence in treatment have restricted the implementation of self-care.	Department of Cardiovascular	patients with chronic heart failure (CHF)	self-care	Intervention group 68.21 ± 4.69 Control group 68.57 ± 4.12	China	2019	Application of self-care based on full-course individualized health education in patients with chronic heart failure and its influencing factors	Sun J, Zhang ZW, Ma YX, Liu W, Wang CY	21
